# A Model for the Origin and Properties of Flicker-Induced Geometric Phosphenes

**DOI:** 10.1371/journal.pcbi.1002158

**Published:** 2011-09-29

**Authors:** Michael Rule, Matthew Stoffregen, Bard Ermentrout

**Affiliations:** 1 Department of Neuroscience, Brown University, Providence, Rhode Island, United States of America; 2 Department of Mathematics, University of Pittsburgh, Pittsburgh, Pennsylvania, United States of America,; Indiana University, United States of America

## Abstract

We present a model for flicker phosphenes, the spontaneous appearance of geometric patterns in the visual field when a subject is exposed to diffuse flickering light. We suggest that the phenomenon results from interaction of cortical lateral inhibition with resonant periodic stimuli. We find that the best temporal frequency for eliciting phosphenes is a multiple of intrinsic (damped) oscillatory rhythms in the cortex. We show how both the quantitative and qualitative aspects of the patterns change with frequency of stimulation and provide an explanation for these differences. We use Floquet theory combined with the theory of pattern formation to derive the parameter regimes where the phosphenes occur. We use symmetric bifurcation theory to show why low frequency flicker should produce hexagonal patterns while high frequency produces pinwheels, targets, and spirals.

## Author Summary

When the human visual system is subjected to diffuse flickering light in the range of 5-25 Hz, many subjects report beautiful swirling colorful geometric patterns. In the years since Jan Purkinje first described them, there have been many qualitative and quantitative analyses of the conditions in which they occur. Here, we use a simple excitatory-inhibitory neural network to explain the dynamics of these fascinating patterns. We employ a combination of computational and mathematical methods to show why these patterns arise. We demonstrate that the geometric forms of the patterns are intimately tied to the frequency of the flickering stimulus.

## Introduction

Ever since they were first described by Jan Purkinje in 1819, the swirling geometric visual patterns brought on by diffuse flickering light have fascinated both scientists and artists. Helmholtz described the patterns at the turn of the twentieth century. The invention of the stroboscope enabled investigators to classify conditions in which they occurred, including, the interactions with hallucinogens. In several papers, Smythies [1], [2] provided detailed accounts of the visual patterns reported by subjects when stimulated over a wide range of frequencies. Knoll [3] studied the interactions between stroboscopic illumination and the hallucinogens, lysergic acid diethylamide (LSD), mescaline, and psilocybin. A concise history of flicker phosphenes along with their influence on the arts is provided in [4]. The recent documentary *Flicker* focuses on the artistic endeavors of Brion Gysin and his *Dream Machine*, a version of a strobe that is powered by a 78 RPM record player.

The first attempts to *quantify* conditions which can produce flicker phosphenes are described in two papers by Remole [5], [6]. These showed that there is a range of frequencies between 10 and 40 Hz in which geometric patterns are perceived. Remole looked at the perception as a function of the luminance and frequency and found a peak sensitivity at 15–20 Hz. He also studied how the patterns depend on the color of the light. Recently, Becker and Elliott [7] revisited this work but, in addition, included subjective descriptions of the patterns and their frequency dependence. Figure 3 in [7] of their paper depicts histograms for the occurrence of patterns as a function of the frequency. At 20–30 Hz, their subjects report spirals, waves, radials (targets), and lines. At 10 Hz, zigzags, honeycombs, and rectangles are reported. In most cases, the different classes of patterns are reported over a broad range of frequencies. Billock and Tsou [8] discuss pinwheels and targets induced by flicker in human subjects by stabilizing the patterns with a small low-contrast “seed” pattern at the center of fixation. They quantified spatial aspects such as the number of spokes on the pinwheels. Allefeld et al [9] sweep through a range of frequencies from 1–50 Hz and record subjective impressions from subjects. They find that subjects have a fairly stable range of frequencies at which they report subjective patterns and that within subjects, the form of the patterns is consistent. A recent review [10] provides a comprehensive summary of the literature on geometric visual hallucinations including a large section on flicker phosphenes.

Based on earlier models of hallucinations [11]–[13], we suggest that the simplest geometric patterns during flicker have their origin in primary visual cortex. Herrmann [14] recorded visually evoked electroencephalograms of subjects exposed to flicker from 1–100 Hz and found strong resonances at 10,20,40, and 80 Hz. Herrmann also remarks that at some of these resonance, subjects report geometric hallucinations. The work of Ermentrout and Cowan [11] was the first to suggest that patterns perceived during the early stages of drug-induced visual hallucinations were a consequence of a loss of stability of the excitatory and inhibitory network comprising the primary visual cortex. This work has been generalized to include other patterns by Bressloff and collaborators [12], [13]. Dahlem and Chronicle [15] created computational models of spontaneous cortical patterns in the context of migraine auras while Henke et al [16] study stationary and moving patterns of activity in a cortical population model. There have been only a few attempts to explain flicker patterns. Knoll [3] describes a vague model that seems to be related to resonance. Stwertka [17] reviews the literature on flicker phosphenes and proposes that they can be viewed as “dissipative structures.” That is, they arise as spontaneous patterns formed through bifurcations and instabilities of the cortical network. However, there was no specific model or mechanism proposed in this review. Drover and Ermentrout [18] describe a model for a periodically driven neural network which is capable of producing slowly evolving line-like contours. These patterns were presumed to reside in the retina (rather than in the cortex) and require, in addition to the periodic drive, an additional transient stimulus. Wilson and Cowan [19] show period-doubling (called “frequency demultiplication” in their paper) in the Wilson-Cowan equations when stimulated at 40 Hz, but did not mention any spatial effects.

Our goal in this paper is to propose a computational and theoretical model for the spontaneous formation of geometric patterns in the presence of flickering light. We first propose a model for a spatially distributed network of excitatory and inhibitory neurons where each neuron is represented by its firing rate [19]–[21]. We simulate one- and two-dimensional (in space) versions of the network and demonstrate that patterns are found only at specific frequencies. We examine the global dynamics of a small network and show dynamics and bifurcations similar to those in the full spatially distributed systems. We next analyze the dynamics of the model by studying the linear stability. We use methods from Floquet theory to compute the boundaries in frequency-contrast space for which there are patterns. We use symmetric bifurcation theory to then explain why some patterns are seen at low frequencies and others at high frequencies. We then discuss some generalizations of the present model toward more realistic networks and stimuli. We close with a discussion of the relationship of these patterns to other types of pattern formation and how to experimentally test some of the ideas.

## Materials and Methods

We utilize a variant of the Wilson-Cowan equations [19], [20] to simulate the effect of flicker on a spatial neural network. The general model takes the form:
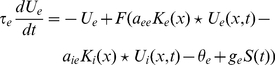
(1)

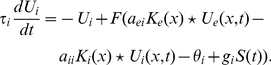
(2)



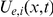
 is the activity of a population of excitatory (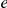) or inhibitory (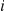) neurons at a spatial location 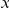. (Note, that this is often erroneously called the firing rate; the 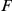 in the equation is the firing rate so that 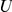 is the low-pass filtered firing rate or “activity”, see [22]) 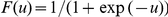 is the conversion factor from input to firing rate of the population. 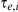 are the time scales of the excitatory and inhibitory activity. The parameters 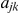 are the maximal connection strengths from population 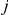 to population 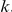 The notation 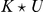 denotes a spatial convolution of 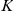 with 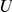 in order to include coupling between neighboring units in one and two spatial dimensions. The domain is either a line segment (in one dimension) or a square in two-dimensions. We take:
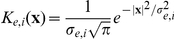



For simplicity, and to avoid edge effects, in our simulations, the boundary conditions are periodic. For most of the paper, we fix parameters to be 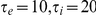, 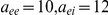, 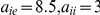, 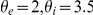, 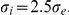 The stimulus has the form
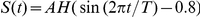
where 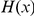 is the unit step function, 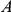 is the magnitude, and 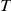 is the period in milliseconds. Time constants 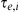 are also in milliseconds. For some of the numerical bifurcation and stability analysis, we make 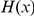 a smooth approximation of the step function, 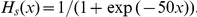 Parameters for the equations are chosen so that in absence of the stimulus, there is a single asymptotically stable equilibrium point for both the full spatial model and for the homogeneous equations. In the latter case, the stable equilibrium exhibits damped oscillations with a frequency of about 13 Hz. The choice of parameters is not arbitrary and the 13 Hz damped oscillations play a crucial role in the emergence of pattern formation. We remark that this frequency is in the range of the scintillation rate of migraine headaches [23], a pathology that is often associated with spontaneous phosphenes.

In the last section of the results, we couple two such two-dimensional networks to represent the left and right hemifields of the visual cortex. Coupling is achieved as follows. Let 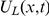 and 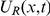 denote the excitatory activity of the left and right networks. Then terms like
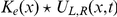
are replaced by
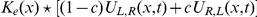



When 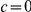 the two are uncoupled.

Analysis of the linearized equations about the oscillatory homogeneous state (for 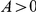, the homogeneous state is not constant), is performed by numerically solving for the monodromy matrix and using this to determine stability (see Results section). We ultimately use continuation with AUTO [24] (implemented within [25]) to compute stability diagrams which are compared to the simulations.

## Results

### Patterns and their transformations

**Figure 1:**
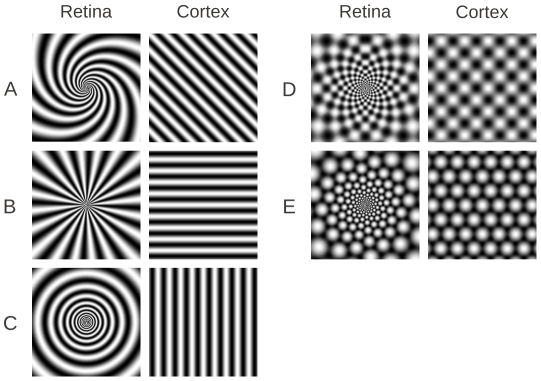
Illustrations of basic phosphene patterns and their transformation to “cortical coordinates.”

The phosphenes reported by subjects vary tremendously, but among them are the commonly seen so-called *form constants* (Klüver, 1960), which are simple regular geometric patterns. These include spirals, targets, light rays, honeycombs, and checkerboards. Figure 1 illustrates idealized versions of many of the reported patterns during flicker stimulation. Figures 1B,C are very typical and are the phosphenes reported by [8] when the visual system was stimulated at 15 Hz as well as by [26] over a range of frequencies between 15 and 20 Hz. Spirals (A) and honeycombs (possibly figure 1E) were also reported in this frequency range. “Rectangles” (possibly interpreted as the checkerboard pattern, (D)) were reported to occur at lower frequencies (around 10 Hz).

Remole [6] quantified the appearance of flicker patterns as a function of both frequency and magnitude of the stimulus. He was rather nonspecific about all the patterns but does mention “clusters of geometric shapes arranged like honeycombs.” He states that the patterns that emerge from binocular stimulation could be “subdivided further in terms of geometric characteristics”, but does not specify them. However, he takes quantitative data from three subjects over a range of frequencies from 5–40 Hz. He plots the minimum luminance required to elicit a pattern for these frequencies. In two of his subjects, there is a single minimum value for the threshold with binocular stimulation at about 20 Hz. The third subject shows two threshold minima,one at 10–11 Hz and the other at 24 Hz.

There is a well-known topographic mapping from retinal coordinates to cortical coordinates ([27] p129) that is roughly the complex logarithm. That is, a point 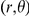 in polar coordinates on the retina is mapped to 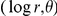 in Cartesian coordinates in the cortex. This means that, for example, the target in figure 1C perceived on the retina is mapped to a series of vertical stripes in the cortex. The other patterns in figure 1 are similarly mapped to simple doubly periodic patterns in the cortex. Ermentrout and Cowan [11] and later Bressloff et al [13] used this same argument in order to explain visual patterns during mescaline hallucinations. Thus, our goal in the remainder of the paper is to determine the types of patterns that are expected in one- and two-dimensional domains during flicker. The main consequences of this topographic mapping can be summarized as follows: (i) target patterns appear as vertical stripes in cortical coordinates, (ii) pinwheels appear as horizontal stripes, (iii) spirals as diagonal stripes, and (iv) honeycomb/hexagon/checkerboards appear as distorted versions of themselves. Thus, for example, if there are vertical stripes of activity on the cortex, the subject will perceive a target with finer structure near the fovea.

### Simulations in one-dimension and two-dimensions

**Figure 2:**
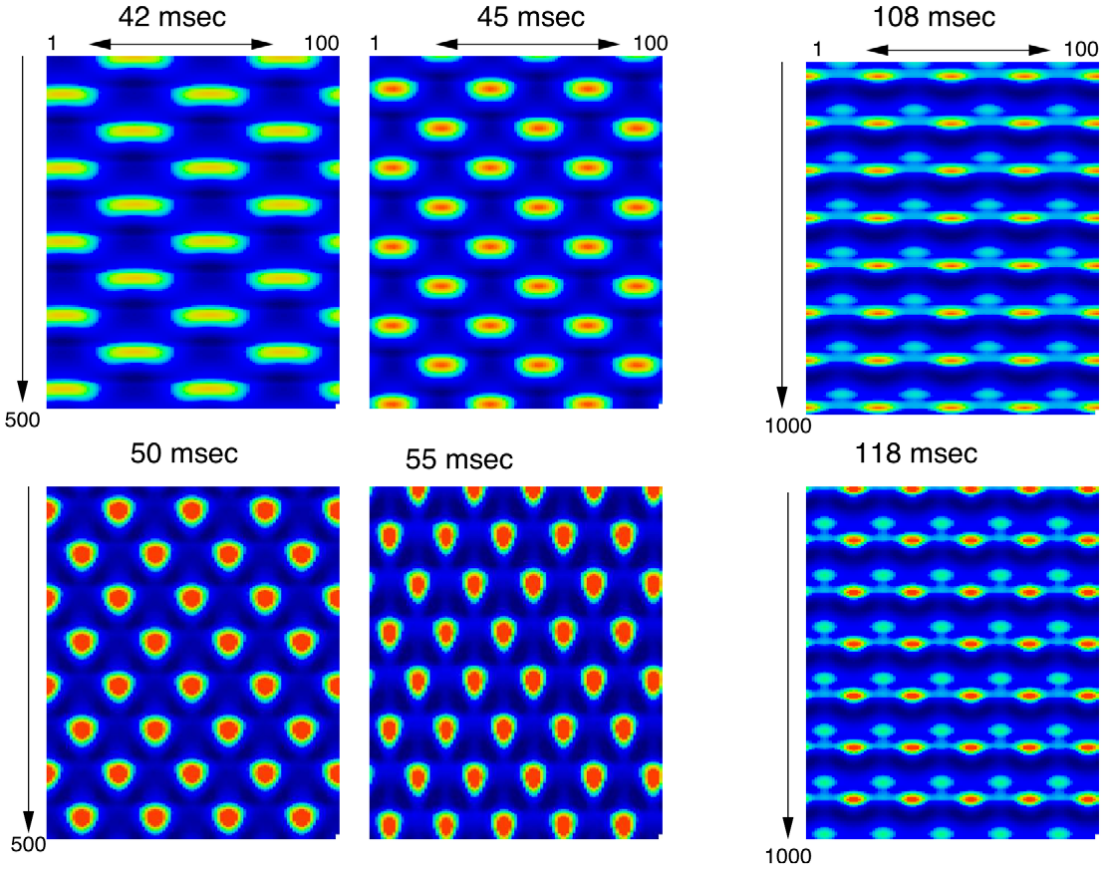
Space-time evolution of patterns produced by **equations (1**,**2**) in a one-dimensional spatial domain. 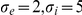
, with periodic boundary conditions. Time increases in the vertical direction. Amplitude of the stimulus was 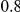 and the period of the stimulus is shown above each panel.

We begin with simulations of a one-dimensional domain since it is much easier to visualize the spatio-temporal dynamics. Figure 2 shows example simulations when the excitatory population is stimulated by periodic pulses of fixed amplitude but varying period. At high frequency stimuli (periods between 40 and 60 msec), the medium breaks up into standing oscillations in which a population at any given spatial location fires on every other cycle. Note that the pattern on one cycle is shifted half a spatial cycle on the next temporal cycle. The overall spatial frequency *increases* with the temporal period within the high frequency region. That is, higher frequency temporal stimuli yield lower frequency spatial responses. The patterns seen here are for a periodic spatial domain; other “boundary conditions” produce similar patterns. The right panel shows two patterns with low frequency stimuli. The patterns show similar spatial dependence in that as the period increases, within this range of long period forcing, the spatial frequency of the pattern increases. More, importantly, the simulations show an important qualitative difference between low and high frequency forcing. For high frequency (short period) forcing, the network responds with a period that is twice the forcing period. Furthermore, there is a clear symmetry in that after one cycle, the background is the foreground and vice versa. For long period (low frequency) forcing, no such symmetry exists and the network responds in a 1∶1 fashion with the stimulus. The difference in symmetries between the two responses to forcing has important consequences for the two-dimensional model as we will next see.

**Figure 3:**
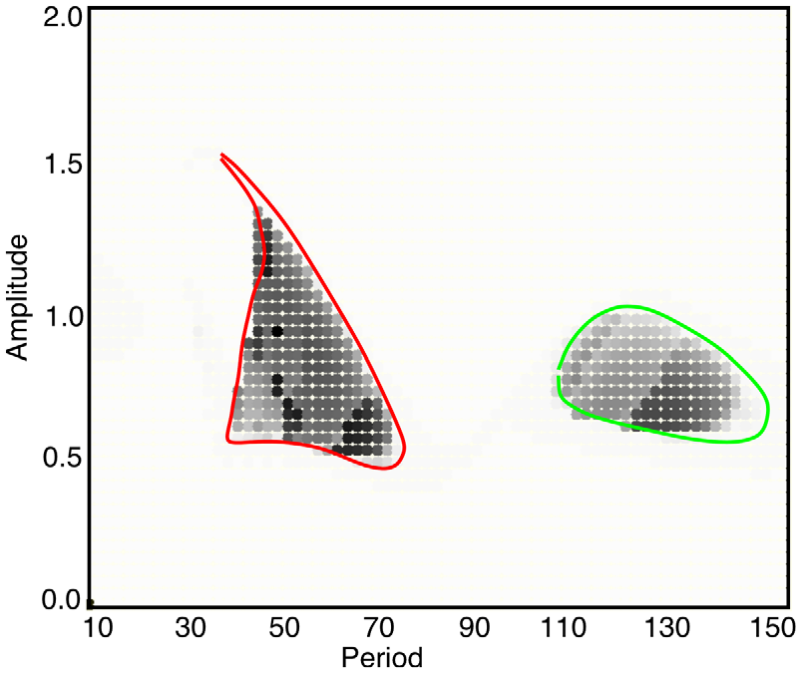
“Phase-diagram” for the one-dimensional spatial model. Each point represents a simulation at a fixed value of the amplitude and period for the flashing stimulus. The gray-scale represents the magnitude of the pattern averaged over time. Specifically, the time average of 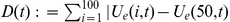 is plotted. If there is a spatially homogeneous pattern, 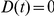. The curves super-imposed on the diagram represent regions where the homogeneous state is shown to be unstable.

Figure 3 shows a phase-diagram for the dynamics of the one-dimensional network. The gray scale shows the quantity
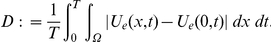



If 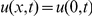 for all 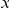, there is no pattern and 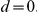 The time window, 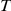, is chosen to be sufficiently long so that many cycles are averaged. There is a limited region for which pattern formation takes place which takes the form of two islands: a low-frequency (long period) cluster and high-frequency (short period) cluster.

**Figure 4:**
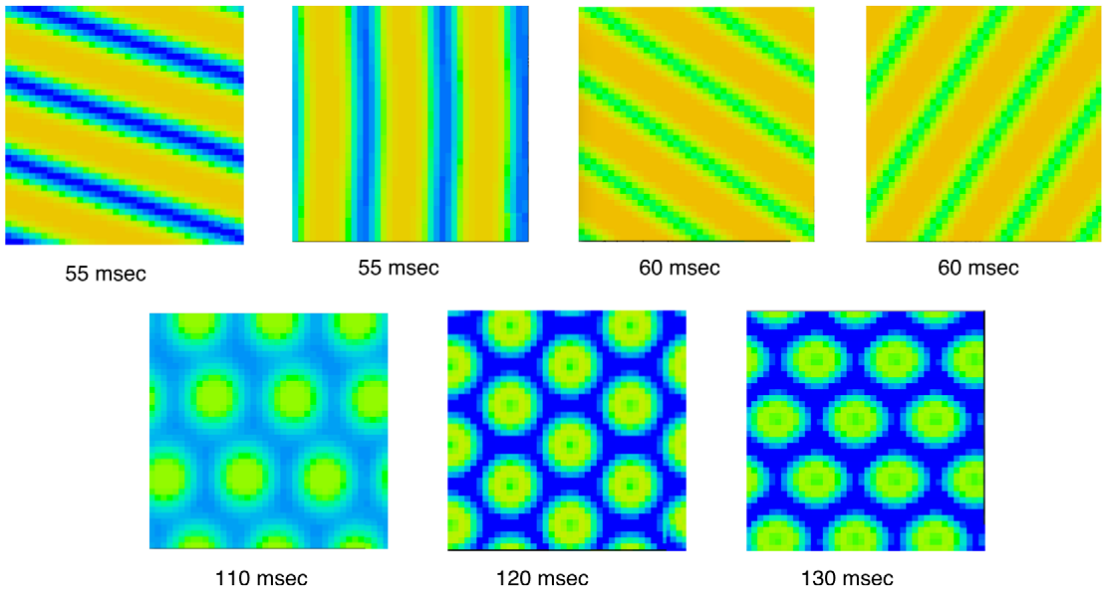
Sample two-dimensional patterns seen in a 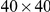 grid with periodic boundary conditions. Top row shows patterns seen with high frequency stimuli. Pairs show the results of different random initial conditions. Bottom row shows patterns seen at lower frequency; each pattern has the same period as the stimulus.

Two dimensional simulations reveal some striking differences. Figure 4 shows patterns seen in a simulation on a 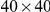 grid. The top row contains examples with a period of 55 and 60 msec. Unlike the one-dimensional simulations, there is multi-stability. For example with a 55 msec stimulus, vertical, diagonal, and horizontal (not shown) stripes are all possible patterns (corresponding to target, spiral, and pinwheel perceptual patterns). Similarly, at 60 msec, two types of diagonal stripes appear. Like the high frequency one-dimensional patterns, the two-dimensional simulations also have a period that is twice that of the forcing stimulus. After one cycle of the stimulus at 55 msec, the left upper pattern looks exactly the same except they are shifted by one half of a spatial cycle so that the yellow background is now the blue foreground and vice versa. The pattern is thus a standing wave in which the foreground and background are perfectly symmetric and alternate with each stimulus. The alternation between the stripes would possibly be perceived as motion and thus, we speculate that what would be seen is an expanding or pulsating target pattern (for horizontal stripes) or a rotating or possibly rocking pinwheel (for vertical stripes). In almost all simulations, we see stripe-like patterns with high-frequency stimuli. This facet of the model is compatible with the psychophysical observations of [7] as well as [8], [28]. We also see that the spatial frequency of the pattern with a period of 60 msec is higher than that with a period of 55 msec as was seen in the one-dimensional models.

The lower row of figure 4 shows time slices of the pattern at low forcing frequencies. Unlike the high-frequency stimulation, the pattern has exactly the same period as the stimulus. That is, each spatial point fires in a 1∶1 manner with the stimulus. The patterns seen are almost always hexagonal and the foreground and background are not simple spatial shifts of each other; they are distinctive patterns. The perception would be like figure 1E (left) where the foreground and background pulsate on and off alternately. Finally, the larger period stimuli produce patterns with higher spatial frequency. Smythies [29] reported a result that is opposite our simulations (lower frequencies gave him coarser patterns), but this result has never been replicated. In sum, the simulations show that at low forcing frequencies (in the range of 8–12 Hz), the patterns are primarily hexagons.

**Figure 5:**
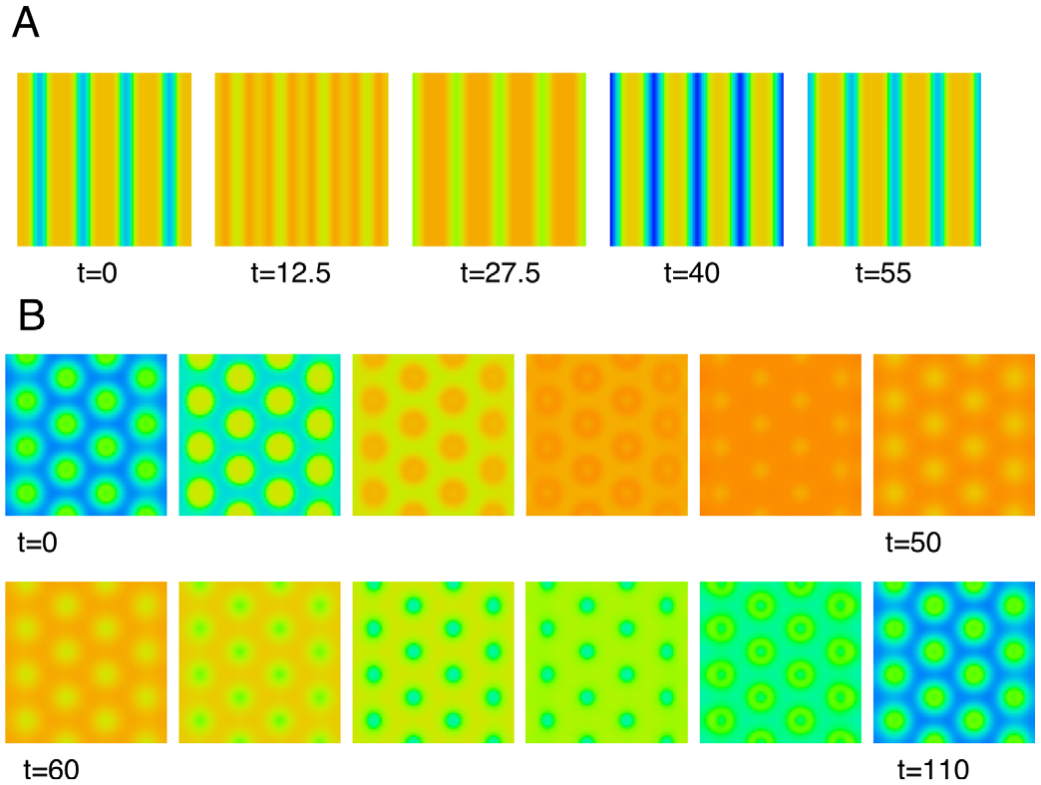
Time frames for different frequencies of stimulus. (A) high frequency stimulation (18.2 Hz); (B) low frequency (9.1 Hz) stimulation. Note that in (A) after one temporal cycle of 55 msec, the pattern is shifted by one half of a spatial cycle.

Figure 5 shows frames from a simulation at various time points over one cycle of stimulation. In 5A, the period is 55 msec (18.2 Hz) and after one cycle of 55 msec, the pattern activity is shifted by one half of a spatial cycle. Thus, the whole cycle of firing takes 110 msec or double the forcing period. In contrast, the simulation in figure 5B shows a period identical to that of the forcing stimulus. However, there is no interchange of the background and foreground like there was in panel A.

**Figure 6:**
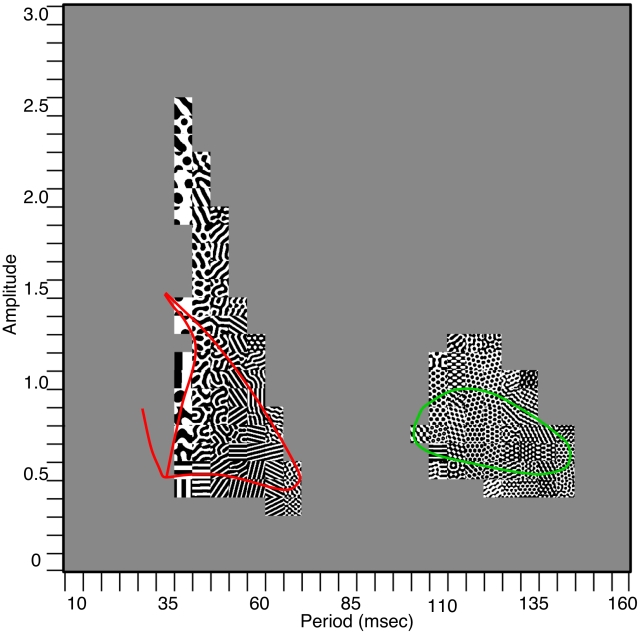
Two-parameter phase diagram for the model. Each square is a simulation of a 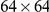 domain with periodic boundary conditions. Patterns are shown as the period and amplitude range over the relevant intervals. Colored curves represent the theoretical boundaries for instability of the spatially uniform state.

Figure 6 shows a two-parameter phase-diagram analogous to figure 3. Each small square is a simulation of a 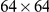 network forced at an amplitude given by the vertical coordinate and period given by the horizontal. As with one spatial dimension, there are two islands of pattern formation. In the short period (high frequency) island, most of the patterns are stripe-like (including labyrinthine patterns) while in the long period (low frequency) island, the patterns are dominated by hexagons.

In sum, the simulations show (i) high frequency stimulation tends to lead to stripes; (ii) low frequency tends to lead to hexagonal patterns; and within each frequency band, the higher frequencies have coarser spatial structure. We lastly remark that the two different regimes are reminiscent of Remole’s observations that one subject had two resonance regions at periods of 90 msec and 42 msec. Our goal in the remainder of this paper is to better understand the reasons for these observations.

### Global dynamics for a highly reduced model

Before turning to the analysis of the spatially distributed domains, we first consider a very reduced system. Suppose that there are two E-I pairs:
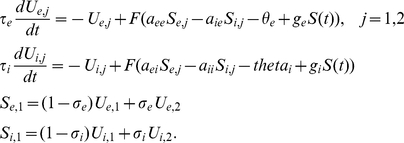
(3)


**Figure 7:**
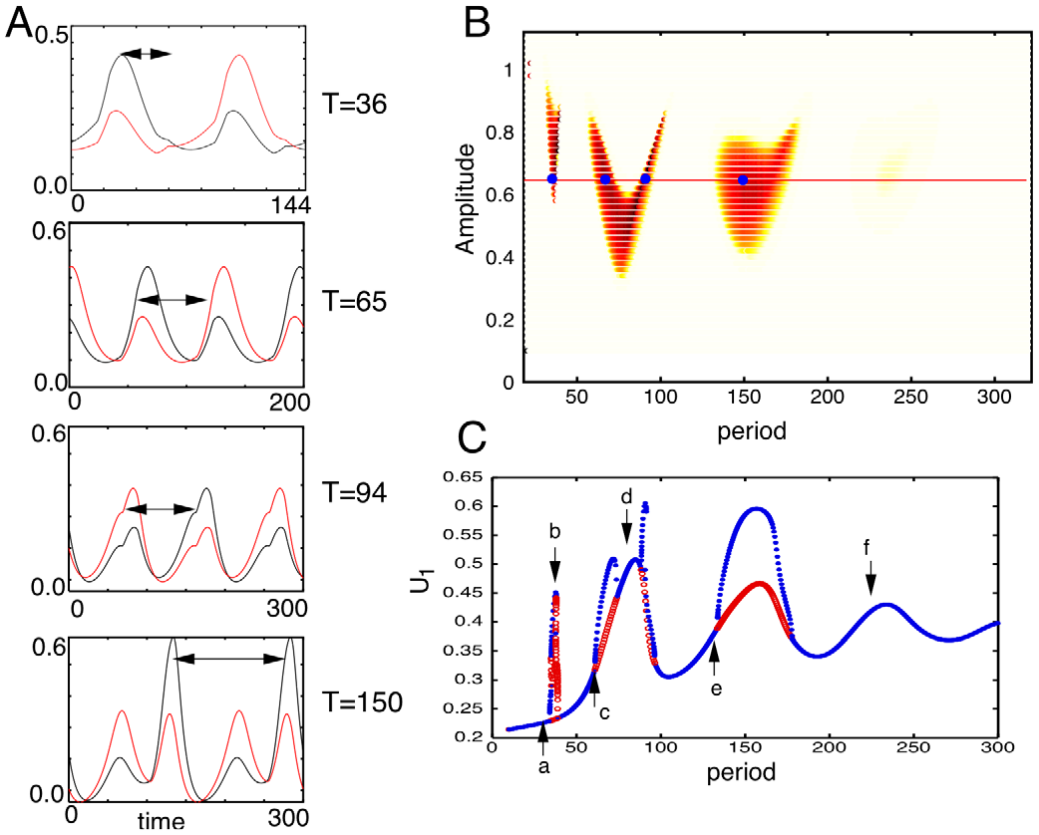
Global picture for the simple 4-dimensional model. (A) Plots of 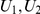 for different periods of forcing with an amplitude of 0.65. (B) Phase diagram as the amplitude and period of the forcing vary; color code is the “depth” of the pattern (the difference between 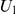 and 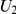) with dark red/black the deepest. (Blue dots correspond to the four periods shown in A.) (C) Bifurcation diagram for 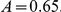 Red curves are unstable periodic orbits and blue are stable. See text for details.

We assume similar equations for 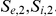 Note that the parameters 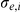 lie between 0 and 1 and determine how strong the interactions between the two pairs are. They are the analogs of the spatial coupling in the one- and two-dimensional networks. Using exactly the same parameters as in the spatial models and with 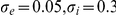, we can perform a similar numerical analysis. Figure 7B shows the phase-diagram for this system as the amplitude of the stimulus and the period vary. As with the spatial models, there are discrete regions where patterns occur. The phase diagram is created by integrating the dynamics forward in time and thus provides only the stable dynamics for a particular initial condition. To get a better picture the full dynamics, we fix the amplitude at 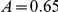 and vary the period using AUTO to continue the periodic orbit. The red line in panel B is a fixed amplitude slice through the phase diagram in which only the stimulus period varies. At this value of 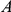, we see that the red line passes through four regions. (The second and third region are part of a contiguous part of the phase-diagram.) We start at the high-frequency (low period) 25 Hz (40 msec) stimulus where equation (3) respond in a synchronous 1∶1 manner. We use AUTO to continue this solution as the frequency decreases (period increases). Figure 7C shows a summary of the numerical continuation of these periodic orbits. The first bifurcation at 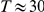 (marked a) results in a period doubling bifurcation of the symmetric solution; that is, both networks fire synchronously. This period doubled solution then becomes unstable through an anti-symmetric period doubling bifurcation (marked b) resulting in a patterned state in which the two networks oscillate out of phase. The whole cycle is four times the period of the stimulus. Figure 7A1 shows the trajectory of the two excitatory cells at a typical point in this parameter regime. The forcing period is 36 msec, but the full cycle is 144 msec. As the stimulus period increases, this pattern disappears through another period doubling bifurcation which again joins with the period one symmetric solution. The next pair of instabilities occur at the points labeled c and d in figure 7C and arise as a period-doubling bifurcation of the symmetric period one state. Unlike the first period doubling bifurcation (at point a), both of these are anti-symmetric and lead to the patterned state in which each unit has the same temporal dynamics that is twice the forcing period and shifted by a half cycle. Figures 7A2,3 show the temporal profiles of 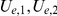 which are just one forcing period shifts of each other. Finally, at the longest periods there is a bifurcation (marked e) to a patterned state that is not symmetric and occurs at a +1 Floquet multiplier. In this pattern, as seen in Figure 7A4, one unit is suppressed and the other active. As this system is symmetrically coupled, the bifurcation at point e is a pitchfork and the other branch of the pitchfork is a state in which 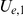 is suppressed and 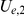 dominates; in other words, the red and black curves are reversed in figure 7A4. The phase diagram, figure 7B indicates a weak pattern at 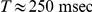, but this is not evident in the bifurcation diagram in panel C. At the point labeled f in panel C, the Floquet multiplier is very close to -1, but remains inside the unit circle. Thus, the synchronous state is stable but weakly so. The apparent pattern in panel C for 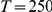 is most likely an artifact of the numerical integration of the equations.

In sum, even with as few as two units, the overall dynamics is qualitatively similar to the full spatially extended networks. The shape of two-network phase-diagram differs from that of the spatially extended network. This is due to the fact that the full spatial system has an infinite number of eigendirections, compared to just the two for the reduced model and that the ratio of inhibitory to excitatory coupling is slightly different.

### Stability analysis

We now want to understand the mechanism for these patterns and to better quantify the dependence of the patterns on the stimulus period. To do this, we next show how to compute numerically boundaries for pattern formation as the frequency and amplitude of the flashing light change. The analysis holds in any dimension and in many types of domains as long as certain conditions are met. We describe the approach generally for 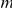 populations of neurons. (For this paper, 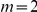, excitatory and inhibitory.) We write the system of equations as
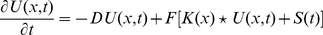
(4)where 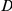 is the diagonal matrix of the reciprocal time constants, 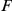 is the vector of firing rate functions and 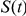 is the vector of spatially uniform stimuli. 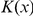 is a matrix of connectivities with
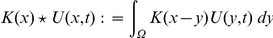
where 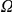 is the spatial domain. In one-dimension, the domain is a circle (periodic boundary conditions) and in two-dimensions, it is a square with periodic boundary conditions. We assume several important properties of the interactions: (a) homogeneity and (b) common eigenspace. Homogeneity means that the network is such that if 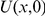 is independent of 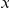, then 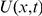 is independent of 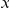 for all time. This just means that spatial homogeneity is preserved. (Note that this does not mean that it is necessarily stable.) The second condition means that there is a set of scalar linearly independent eigenfunctions, 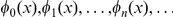 such that for each of the component entries, 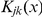 that constitute the matrix 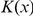, we have
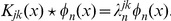



For example, if the domain is the circle (that is periodic boundary conditions in one dimension), then 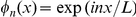 where 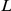 is the circumference of the circle and if the domain is the 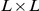 square with periodic boundary conditions, the eigenfunctions have the form 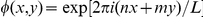. We also assume that the eigenvalues, 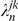 are real. Since we have assumed homogeneity, 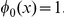 We define 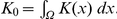 The spatially homogeneous network satisfies:
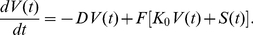
(5)


This is a nonlinear periodically forced system, so we are not guaranteed that there is a periodic solution. Let 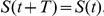 We make our final assumption: there is a 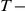
periodic solution 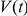 to equation (5). Notice that a solution to (5) is automatically a solution to the full spatial problem, (4) by our assumptions of homogeneity. To understand pattern formation, we linearize equation (4) about the homogeneous solution 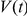: 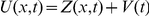 where 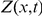 is the infinitesimal perturbation from the homogeneous state. The linearized equations for 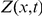 satisfy
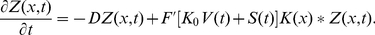
(6)


We now invoke our hypothesis about eigenfunctions. We write
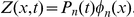



If we plug this into (6), we see that
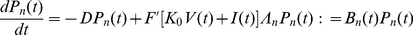
(7)where 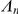 is the matrix of eigenvalues 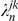 and 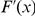 denotes the derivative of 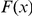 with respect to 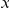. We have reduced the stability question to the study of a system of linear differential equations with periodic coefficients. Of course, there are an infinite number of these equations, one for each 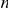. However, for reasonable functions 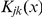, 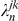 rapidly go to zero, so that 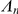 will be close to zero and thus, solutions to (7) will decay like 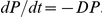 In practice, therefore, we need only worry about a finite number of 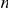 values.

The way to solve a linear equation with periodic coefficients is to compute the so-called monodromy matrix. Let 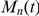 be the matrix solution to
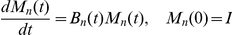
where 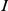 is the identity matrix. Compute this for one period to get 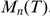 This matrix is called the monodromy matrix. A general result from the theory of linear periodic systems is that solutions decay to zero if and only if all of the eigenvalues of 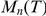 lie inside the unit circle. Since 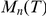 is 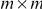 (there are 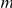 populations), there will be 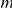 eigenvalues for 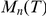, 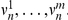 For large 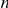, 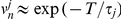 where 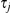 is the time constant for the 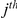 population.

For our system, 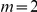 and 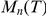 is just a 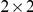 dimensional matrix. Eigenvalues satisfy 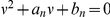 where 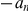 is the trace of 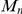 and 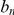 is the determinant. Thus, we need only study these coefficients to determine stability. There are three qualitatively different ways that an eigenvalue can exit the unit circle: 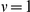, 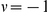 and 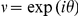 where 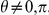



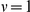
 when 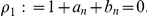 When this occurs, we expect to see a pattern that has a spatial shape like 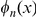 and that has period 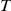, the same as the forcing period. The low frequency pattern with period 110 msec is such an example. When 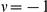, then 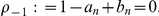 This leads to a period doubling bifurcation; a pattern arises that has period 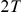 alternating between 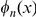 and 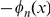; that is, what is the foreground in one cycle is the background in the next. The pattern with period 55 is such an example. Finally, when 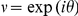, 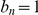 and quasi-periodic, complex periodic, and possibly chaotic solutions and appear. We have not seen this type of instability in our model.

To compute stability boundaries, we need to find and parameterize the eigenvalues, 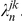 For the models considered here, the spatial interactions are homogeneous so that the eigenfunctions will be spatially periodic and from these we can easily obtain the eigenvalues. We will illustrate this idea for a one-dimensional network on the circle of length, 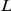 and in a two-dimensional 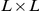 square domain with periodic boundary conditions. While we sometimes simulate on domains that are not periodic, for a large enough domains, the patterns and eigenfunctions will look very similar. For a one- (two-) dimensional periodic domain with length 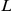, the spatial eigenfunctions have the form 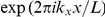 (respectively, 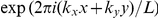) and the convolution operator has eigenvalues, 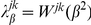 where 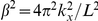 (respectively, 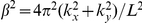). Here 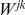 is the Fourier transform of the spatial weight functions, 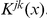 We see that in both one and two spatial dimensions, the eigenvalue can be parameterized by a single variable, 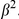 For 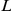 large, as 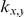 range through the integers, 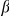 fills in nearly a continuum of numbers. Thus, we replace 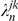 in equation (7) by the continuous parameterization, 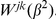 Now we just range through 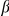 and look for stability boundaries. Suppose there is a value, 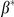 such that an instability is reached. Then this value will be close to 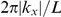 (respectively 
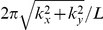
) for some integer 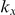 (respectively, pair of integers, 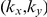 ) and this will determine the spatial patterning.

For our system, we have used Gaussian spatial interactions with space constants, 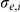 for the excitatory and inhibitory neurons, thus, 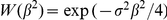 so that we need to solve equation (7) with
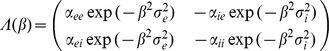



**Figure 8:**
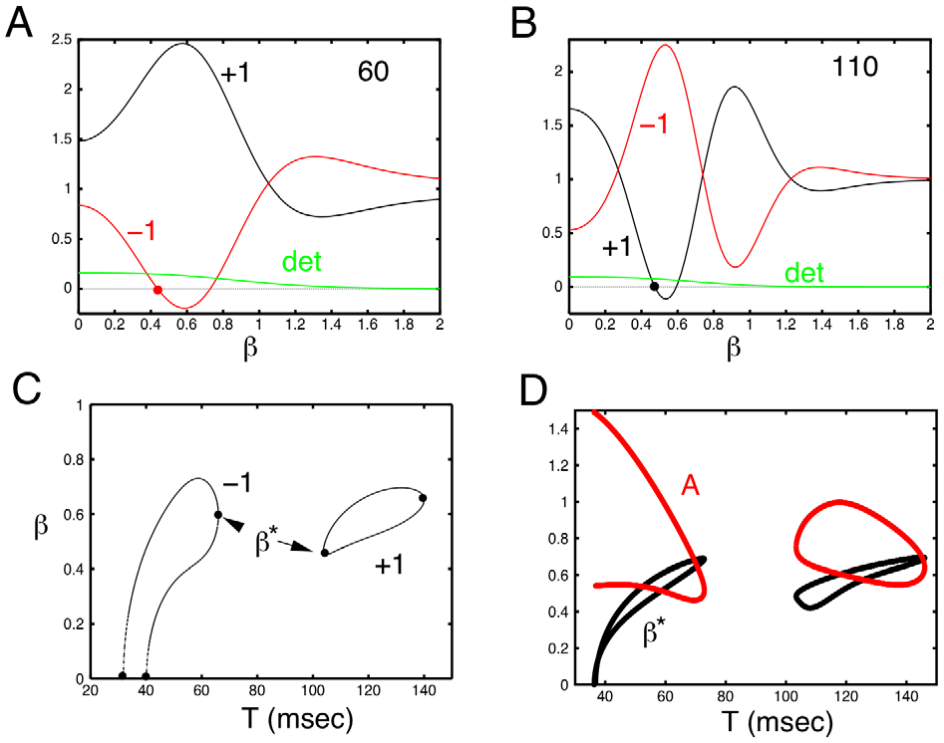
Stability calculation illustrated. (A,B) Values of the determinant (green) and stability conditions for a +1 (black) and −1 (red) Floquet multiplier as the wave-number, 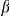 varies. For stability the determinant must be less than 1 and the black and red curves should be positive. (A): 60 msec and (B): 110 msec forcing period. (C) Wave numbers, 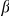 where the 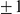 curves from A and B cross the zero axis as the forcing period varies. Black dots correspond to local extreme values delineating the edge of stability regions. Inside these domains the spatially uniform oscillation is unstable. (D) Critical region for stability as the amplitude and period vary. Red curves correspond to the amplitudes, 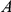 and black to the critical wavenumber, 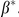

Figure 8 shows an example of the stability calculation for two different forcing periods, 60 and 110 msec. In each of the plots A,B, three curves are plotted, 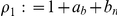 (in black), 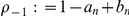 (in red) and 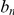 (in green). The eigenvalues of the monodromy lie in the unit circle when 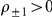 and 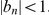 Then 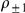 becomes negative this means that an eigenvalue of the monodromy matrix crosses 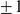 so that the homogeneous state becomes unstable. Thus, black (respectively, red) curves crossing zero lead to +1 (respectively, −1) eigenvalues. In the 60 msec example (panel A), as 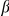 increases, we see that the red curve that corresponds to a 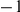 eigenvalue crosses zero for 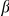 between 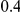 and 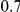. In panel B, when the period is 110 msec, the loss of stability occurs through a 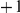 eigenvalue at 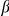 between 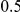 and 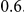 Since 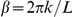, for 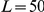, we compute 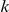, the wavenumber, to be between 3 and 5 which is close to the value seen in the simulations in figure 4 top.

Once we have found an intersection of one of the curves, 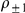 with zero, we can then follow that zero using AUTO as a function of the period, 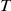 of the stimulation. Figure 8C, shows two curves in which we trace the zeros of 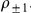 (For example, a vertical line at 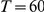 intersects the leftmost black curve, 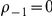 in panel C and this corresponds to the two zeros of the red curve in panel A at 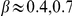.) If we change the period slightly, then the curves in panel A will look somewhat different. At some critical value of the period, 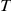, in panel A (respectively panel B), the red curve, 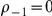 (respectively, the black curve, 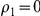) will be tangent to the 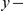
axis. This occurs at the point 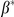 shown in panel C. We now follow this tangency as we vary the amplitude, 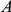 of the stimulus producing the two-parameter diagram shown in figure 8C.

We can understand figure 8C as follows. Suppose that we fix the magnitude of the stimulus at 0.6. We start flash the strobe at 50 Hz (a period of 20 msec) and slow it down. When it reaches a period of about 40 msec, we enter the enclosed region in the figure labeled -1. Inside this region, the uniform state is unstable and a pattern should appear. Since the transition occurs in the -1 region, the pattern will repeat every 80 msec with the foreground and background alternating. As the frequency continues to decrease (and the period to increase), we leave the curve at about 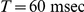 and the uniform state is stable. Continuing to increase the period (decrease the frequency) we run into the second region where there is a +1 instability and again we get patterns. Â However, these patterns repeat with the same frequency as the stimulus. Eventually, we run into the region where no patterns occur and the homogeneous state is stable.

In figure 3, we superimpose on the numerical simulations (in the two colored curves), the stability calculations from figure 8C. There is excellent agreement. Figure 6 shows the analogous diagram for the two-dimensional simulations. The agreement is not as good. We suspect that the main reason that the simulations show a wider range of pattern formation is that the time-step we chose was too large (the simulations are very time consuming, so we took larger than optimal time steps) which then produces numerical artifacts. (The numerical routine is thus solving a discrete dynamical system rather than a continuous one.) We have made more careful (smaller time step) simulations at points near the edges of the colored curves and these show agreement more like is seen in the one-dimensional system.

### Feed-forward inhibition

**Figure 9:**
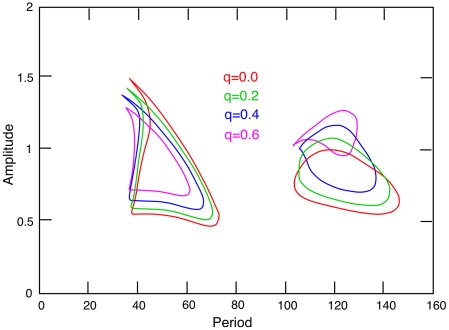
The effects of feed-forward inhibition on pattern formation. Stability boundaries for the homogeneous solution as amplitude and period vary, but the inhibitory population receives input of strength 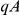 where 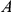 is the amplitude.

So far, the simulations and stability analyses have all been for equations (1–2) when 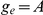 and 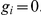 That is, the inhibitory population receives no external stimulation. In figure 9, we redo stability calculations similar to those in figure 3, but we set 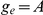 and 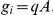 Even for feed-forward inhibition as much as 60% of the excitation, it is still possible to form spatial patterns. The enclosed regions are shifted toward shorter periods (higher frequencies) and toward larger amplitude stimuli. They also have a smaller area indicating that feed-forward inhibition restricts the range of parameters such that patterns are possible.

### Minimal assumptions of the model

In order to get pattern formation we have to make several important assumptions on the local circuit dynamics and the coupling. With no coupling, the “space-clamped” system should have a damped return to a stable rest state. Furthermore, the stable equilibrium should lie on the middle branch of the excitatory nullcline (the so-called “inhibition-stabilized” regime [30]). For our choice of parameters, the equilibrium is a stable spiral and the period of the damped oscillation is about 76 milliseconds. Finally, we require that the coupling implements “lateral-inhibition”, so that the effects of inhibition outreach those of excitation. This assumption is commonly made for pattern forming systems [31].

### Hexagons versus stripes

One of the most striking findings of our simulations is that low frequency stimuli mainly lead to hexagons and high frequency generally lead to stripes. There turns out to be a deep theoretical reason for this result that is based on the ideas of symmetric bifurcation theory. We do not discuss the rigorous mathematics that underlies this theory, but rather, summarize the basic ideas. Near the onset of the instability, the pattern will look like a sum of the eigenfunctions, 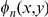. Suppose that the eigenfunctions are of the form
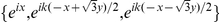
and their three complex conjugates. (This is the minimal set of eigenfunctions which could produce stripes, hexagons, or checkerboard patterns.) We label these three functions, 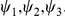 Thus the solution near the bifurcation has the form
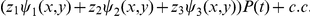
where c.c. means complex conjugates and 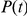 is either a 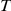 or 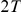-periodic vector function. 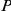 will be 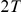 for the high-frequency stimulation and 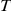 for the low frequency. The linear theory tells us nothing about the coefficients 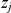. Since we look for patterns that are real, for any term like 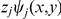,will be accompanied by a term of the form 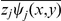, its complex conjugate. If, for example, 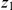 is nonzero and 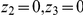, then the pattern will be periodic in 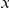, that is, vertical stripes. If 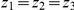, then the pattern will be hexagonal. If, 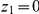 and 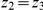 are nonzero, then the pattern will be rectangular. One of the key questions in symmetric bifurcation theory is how to determine what patterns are selected and which are stable. It turns out (see [32], page 151), that the three complex amplitudes, 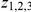 generally satisfy
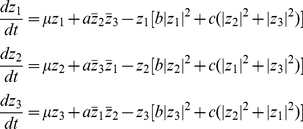
(8)where the real numbers 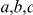 depend on the nature of the equations and 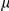 is the deviation of the bifurcation parameter away from the critical value. That is, suppose that the stimulus amplitude is say, 0.4 and the period of the stimulus increases from 20 msec. As seen in figure 8C, at 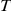 approximately 30 msec, the uniform oscillation loses stability. 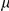 characterizes how far away and in which direction you are from the critical stimulus frequency.

**Figure 10:**
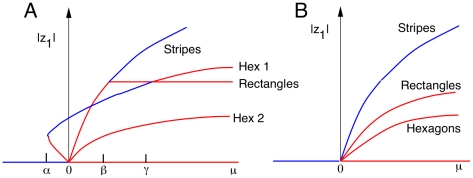
Schematic bifurcation diagram for patterns on a hexagonal lattice. (A) Bifurcation at low frequencies in equation (8) for the case where 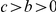 and 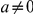 (after [32], figure 5.8). 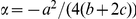, 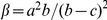 and 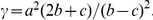 Blue curves represent stable solutions and red curves represent unstable solutions. The bifurcation parameter 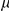 is along the 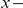
axis and the magnitude of the component of vertical stripe pattern (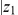) is along the 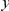-axis. (B) Diagram for high frequency patterns where 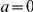 by symmetry. Because 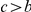, only stripes of various orientations are stable.

Figure 10 shows a schematic bifurcation diagram for equation (8) in the case where 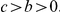 Figure 10A shows the case when 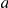 is nonzero. An unstable branch of hexagons (labeled Hex 1) emerges for 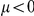 and at the point 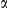 turns around to become stable. This means that there are hexagons that are stable even for 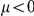, that is, for parameters when the homogeneous rest state is stable. Thus, as we change the frequency of the stimulus so that 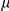 becomes positive (the uniform field loses stability) the network will “jump” to the branch of stable hexagons. Thus, for a range of bifurcation parameters (e.g., intensity and frequency of illumination), for 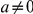, stable hexagonal patterns emerge. Figure 10B shows a diagram for the case in which 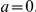 Here, the only stable patterns to emerge are stripes and they always occur when the uniform state is unstable. Unlike the 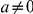 case, there is no multi-stability. Symmetric bifurcation theory tells us one more amazing fact: if the onset of instability is through a 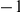 eigenvalue (that is, the case we saw with high frequency stimuli), then 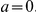 In contrast, if the bifurcation occurs at a 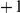 eigenvalue, then, 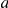 is not generally expected to vanish. Thus, what we can conclude from the nonlinear analysis is that for low frequency stimuli, the first stable patterns to emerge are hexagons. At high frequency stimuli that lead to the so-called period doubling bifurcation, either hexagons or stripes can be stable and it depends on the specific nonlinearities (specifically, whether or not 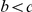) in the model. We have never been able to stabilize hexagons at high frequencies with the simple Wilson-Cowan model described here, so we can conclude that 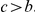 One way to assure that 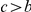 and thus have stripes rather than hexagons bifurcate at high frequency stimuli is to make sure that the resting state of the unstimulated cortex is positioned close to the inflection points of the firing rate function 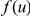 [33], for, at the inflection points, the Taylor expansion of the function 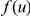 contains no quadratic terms. In sum, at low frequencies where there is 1∶1 firing of the neurons with the stimulus, we always expect hexagonal patterns. At high frequencies, stripes will be more likely than hexagons if we operate near the maximal sensitivity of the firing rate function (near the inflection point). (See also the discussion.)

### Coupled hemifields

**Figure 11:**
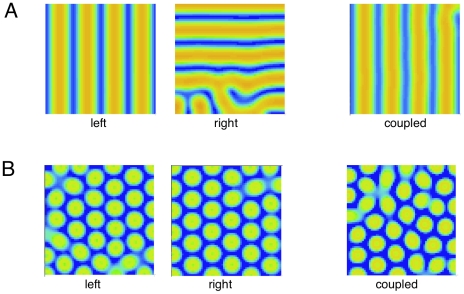
Steady state for 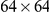 arrays with and without coupling. (A) Period 55 msec without coupling (left two images) and coupled 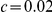 right image. (B) same as (A) but period is 120 msec.

When flicker hallucinations are perceived, they are often seen as whole-field patterns and the patterns are “pure” rather than a mixture of say pinwheels and targets. Thus, a natural question is how can the two halves of the visual cortex “synchronize” their spatial patterns. There is strong anatomical [34] and functional [35] evidence for direct corpus callosal connections between the two halves of primary visual cortex. Thus, we can simulate a pair of such networks with coupling between them. To illustrate spatial alignment, we simulate two square domains where there is reciprocal coupling from a spatial location 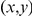 in one domain to the same location in the other. Figure 11 shows both high- and low-frequency examples. In Figure 11A, we have chosen the initial conditions so that without coupling the left and right domains have stripes of opposite orientations. We next restart the simulation but with weak coupling between the two sides and the result is that both sides converge to the same pattern (shown on the right). Figure 11B shows a similar simulation when the stimulus period is 120 msec (low frequency). Without coupling the left and right sides are misaligned, but with coupling turned on, they are exactly the same (rightmost panel). Thus, the coupling both aligns the patterns and forces the two sides to select the same class of pattern (e.g., horizontal or vertical stripes). We want to emphasize that the choice of coupling between hemifields was for convenience and to illustrate the general principle. Indeed, in other simulations, we couple just a thin band of neurons that would be near the “midline” of the cortex. Almost any form of coupling, if sufficiently strong, should lead to the two halves producing identical patterns. The mathematics of this “spatial synchronization” remain to be analyzed.

## Discussion

In this paper, we have suggested a simple mechanism for flicker-induced hallucinations. We suggest that all that is needed is a spatially extended lateral-inhibitory network of excitatory and inhibitory neurons along with some resonance properties such as a damped oscillatory return to the resting state. The lateral inhibition is necessary to produce spatial instabilities as has already been suggested by [11] and subsequently by many other authors [12], [13], [16]. In order to interact with flickering light, there should be an amplification of the activity at certain frequencies. The simplest way to produce this is that the resting state of the network exhibits damped oscillations in the frequency range of about 7–14 Hz (period from 70 to 140 msec). Two types of resonance were evident in our model: 1∶1 resonance where low frequency flicker produces large amplitude spatio-temporal patterns in the 7–10 Hz range; and 1∶2 resonance where individual groups of neurons fire at 7–10 Hz, but out of phase with other neurons producing a pattern where some neurons fire on every cycle. The mechanism for the 1∶2 resonance is mathematically similar to that which produces Faraday waves in periodically forced fluids [36], thus, we expect that the nonlinear analysis follows in a similar vein. Crevier and Meister [37] report period doubling in the human electroretinogram when subjects are exposed to light at 46 Hz, but 1∶1 locking at 26 Hz. Our model shows period doubling at lower frequencies, but we are modeling cortex rather than the retina; the response may be different.

Our model, being based on the earlier models for hallucinations [11]–[13], presumes that the patterns arise in primary visual cortex. Similar structure is found in higher visual cortical areas, but, in these areas, the topographical representation of visual space is much too coarse for patterns such as those in figure 1 to be perceived. ffytche [38] found that V4 was most active during flicker hallucination. This area of visual cortex contains cells that are sensitive to radial patterns such as pinwheels and targets [39] which could be activated by feed-forward connections from V1. Thus, if V1 produces the stripe patterns that correspond to the radial phosphenes in figure 1, these patterns would then excite V4 which could produce the large signal seen in the fMRI data of ffytche. ffytche also found *no increase* in the activity of V1 during flicker stimulation which would seem to contradict the present modeling efforts. However, in our model, the spatio-temporal average of the activity does not change very much during the flicker, rather, it becomes spatially structured with some areas less active than baseline and others more active. The spatial structure of our striped and hexagonal arrays is likely to be too fine to be picked up by imaging. Furthermore, in our model, stripes alternate their activity at roughly 10 Hz, so that any temporal averaging of the signals would completely wash out the pattern and the activity would remain close to baseline.

In order to produce a model that is capable of creating these patterns, the cortex has to be in a particular state. Geometrically, we want the excitatory and inhibitory nullclines of the space-clamped system (the local circuitry) to both have positive slopes at the resting state. In a recent combination of theory and experiment, [30] (c.f. Figure 6D) suggested that the visual cortex lies in a so-called “inhibitory-stabilized” configuration. That is, the inhibition was necessary to overcome the strong recurrent excitation that causes a positive slope in the excitatory nullcline. There are several consequences of this configuration. Unless the inhibition is extremely fast, the return to rest will be accompanied by decaying oscillations. Furthermore, small changes in the inhibition can destabilize the resting state to produce large amplitude synchronous oscillations that could be the analog of seizure activity. Interestingly, there is a strong association with stroboscopic flicker with certain forms of seizure activity, particularly in the range of frequencies that we have studied here. Small changes in the balance of excitation and inhibition could have big effects on the ability to perceive these patterns. For example, benzodiazepines enhance the effects of the inhibitory neurotransmitter GABA, so that we would predict that the enhanced inhibition would reduce the sensitivity of flicker stimuli and result in less vivid phosphenes if perceived at all. Siegel [40] describes a patient whose LSD flashbacks were triggered by flicker, but only after heavy use of caffeine and nicotine. It should be easy to study the thresholds for phosphene generation after use of these readily available stimulants.

There are many generalizations of this model which could be considered. Smythies [29] and Knoll et al [3] study the combination of flicker with hallucinogens and report that the combination of flicker with sub-clinical doses of mescaline can produce phosphenes that are as vivid as those seen with normal doses of the drugs. If we suppose that the action of hallucinogens is to shift the resting dynamics of cortex into an unstable regime [11], say, by changing the threshold of the excitatory population, then we could easily systematically explore the combination of flicker with a shift in the stability.

With very little change in the details of the equations, it should be possible to introduce the “seeding” of patterns into the model. For example, suppose that we are in the low frequency stimulation regime and now add a small bias in the form of say a low contrast target or pinwheel. (In the equations, we would model this as a low contrast grating of the appropriate orientation.) We could then see if the model would produce stripes instead of hexagons as stripes remain a possible pattern. Indeed, the schematic bifurcation diagram in figure 10A shows that stable stripes could be possible when 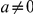 (the low frequency regime). The stability may be shifted toward lower values of 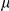 (the stimulus parameter) when such a bias is applied.

Many of the phosphenes reported by subjects are not the broad forms shown in figure 1; rather, they include zig-zags, filigrees and patterns that are much finer. The more general models of Bressloff et al [12] include the equations for the orientation preferences of cortical neurons and produce the fine filigree hallucinations. We expect with some adjustments (such as using a two-population model rather than a single population), we should be able to obtain these more complex patterns with flicker.

An exciting direction to go in this work is to explore the role of color. The phosphenes themselves are extremely colorful. In addition, the color of the light stimulus can have a strong effect on the pattern [5]. The present model does not account for any of the color effects. What is needed is a model that incorporates the color features of the visual cortex. We hope to build such a model in the future.

The emergence of patterns in periodically forced spatially distributed systems has a long history, particularly, in the area of fluid mechanics [41]. Gollub and Langer [42] review pattern formation in parametrically excited granular material and Rayleigh-Benard convection. Crawford [43] was the first to derive equations like (8) for periodically driven surface waves of fluids. Rucklidge [44] analyzes more complex patterns which can arise in the Faraday experiment, including so-called quasi-patterns which are almost, but not quite, regular. (See also [32].) Some of the recent work by Silber and colleagues [36] on two-frequency forcing suggests experiments that could easily be done on the visual system. While the physics of these pattern forming models is completely different from the physics that underlies spontaneous pattern formation in the nervous system, the underlying mathematics is identical. Fluids, granular material, and other physical systems have characteristic time scales which with the right temporal forcing can be excited, just like the pumping of a swing. The spatial patterns which emerge in the physical models are determined by the multiple length scales present. Near the onset of instability all spontaneous pattern formation is governed by a simple set of equations, such as (8), whose form depends on the geometry and symmetries of the particular system.

Flicker stimuli provide an excellent way to probe the intrinsic pattern forming capabilities of the visual cortex since, unlike drug-induced hallucinations, they can be readily controlled. Indeed, [8] have shown that by including a small spatially structured pattern as a “seed” during flicker stimuli, it is possible to stabilize a full-field target or pinwheel pattern. Thus, it may be possible to combine stabilized flicker and brain imaging to see actual hallucination activity in human visual areas.
